# A pilot study to evaluate the application of a generic protein standard panel for quality control of biomarker detection technologies

**DOI:** 10.1186/1756-0500-4-281

**Published:** 2011-08-11

**Authors:** Susan Pang, Enamul S Ahsan, Hernan J Valdivia, Jesus Minguez, Carole A Foy

**Affiliations:** 1 LGC, Queens Road, Teddington, Middlesex, TW11 0LY, UK

## Abstract

**Background:**

Protein biomarker studies are currently hampered by a lack of measurement standards to demonstrate quality, reliability and comparability across multiple assay platforms. This is especially pertinent for immunoassays where multiple formats for detecting target analytes are commonly used.

**Findings:**

In this pilot study a generic panel of six non-human protein standards (50 - 10^7 pg/mL) of varying abundance was prepared as a quality control (QC) material. Simulated "normal" and "diseased" panels of proteins were prepared in pooled human plasma and incorporated into immunoassays using the Meso Scale Discovery^® ^(MSD^®^) platform to illustrate reliable detection of the component proteins. The protein panel was also evaluated as a spike-in material for a model immunoassay involving detection of ovarian cancer biomarkers within individual human plasma samples. Our selected platform could discriminate between two panels of the proteins exhibiting small differences in abundance. Across distinct experiments, all component proteins exhibited reproducible signal outputs in pooled human plasma. When individual donor samples were used, half the proteins produced signals independent of matrix effects. These proteins may serve as a generic indicator of platform reliability.

Each of the remaining proteins exhibit differential signals across the distinct samples, indicative of sample matrix effects, with the three proteins following the same trend. This subset of proteins may be useful for characterising the degree of matrix effects associated with the sample which may impact on the reliability of quantifying target diagnostic biomarkers.

**Conclusions:**

We have demonstrated the potential utility of this panel of standards to act as a generic QC tool for evaluating the reproducibility of the platform for protein biomarker detection independent of serum matrix effects.

## Introduction

Protein biomarkers for diagnosis of disease have formed the basis of clinical research proteomics for several decades [1-3]. In spite of FDA approval of various disease protein biomarkers, including CA-125 for ovarian cancer and prostate specific antigen for prostate cancer, few biomarkers are adopted in standard clinical practices [4]. The FDA highlighted this issue as a major challenge to developing new medicinal products [5]. A key hindrance identified was the lack of assay robustness, which may be improved using appropriate measurement standards and control materials. These reference standards ensure robust comparability of a diagnostic test for the same patient between distinct test sites, or for tests after significant time intervals.

Many protein-based detection methods suffer from a lack of standardisation with the reagents and methods employed [6], in a similar way to microarray assays prior to the advent of the MIAME checklist [7]. With conventional immunoassays, significant variability may exist by using finite sources of polyclonal antibodies which differ in immunogenicity [8]. Variable performance from distinct platforms may arise from differences in reagent quality or platform bias. Commercial immunoassay kits lack standardisation to ensure the traceability of measurements. Often the source or identity (e.g. clone number for monoclonal antibodies) of capture and detection antibodies used in kits are not stipulated [9]. Improved standardisation may be achieved through the use of generic protein standards, demonstrating the reproducibility of the platform function. Such generic standards are emerging for mass spectrometry analysis of proteins, though they are specific to this platform rather than for broad stream applications including immunoassays [10].

For most protein biomarker assays, the diagnosis of diseases may be achieved by detecting the appropriate protein biomarker(s) above specified thresholds, alongside the generic QC proteins to indicate platform functionality. The change in the collective signal output profile of these QC proteins may indicate the presence of inhibitors within the biological matrix, and may infer that the robustness of detection of the target diagnostic biomarker(s) is also adversely affected.

In this paper we have prepared a panel of generic protein standards and evaluated its utility as a quality control (QC) material using the to MSD^® ^platform. The scope of detecting each protein amidst the full panel of proteins was assessed, as well as the ability to identify small known changes in the protein composition. The panel of protein standards was also evaluated as a spike-in material, by supplementing individual donor plasma (ovarian cancer diseased and non-diseased) samples with the QC material. This pilot study revealed the value of the QC material as an indicator of platform robustness, as well as for highlighting any matrix effects associated with individual samples that may influence the reliability of detecting the target analytes within the test samples.

## Experimental sections

### Preparation and storage of the generic panels of protein standards

A generic panel of protein standards was prepared for use as a quality control (QC) material. The composition of this generic panel of proteins (incorporating mouse CCL6 [Uniprot: P27784], mouse lungkine [Uniprot: Q9WVL7], chicken caronte [Uniprot: Q9PUK2], mouse soggy [Uniprot: Q9QZL9], firefly luciferase [Uniprot: Q27758] and chicken egg lysozyme [Uniprot: P00698]) is shown in Table 1. Six non-human proteins that vary in their chemiphysical properties, i.e. size, charge, hydrophobicity and isoelectric points were selected. Sequence comparisons were made between these non-human proteins and their human homologues to confirm sufficient differences in the peptide sequences exist, or in the case of soggy, that there is no endogenous presence of protein in adult human serological samples. These criteria ensure that the proteins are amenable for use as spike material within human serological samples. For the assays, the antibodies raised against each protein were selected on the basis of their specificity to the species of the chosen proteins.

**Table 1:** The components and parameters of the proteins within the spike panels

Non-human Protein (with UniProt accession number)	Molecular Weight (kDa)	pI	Purity (%)	Spike concentration (pg/mL)	
				
				Normal panel of generic standards	"Simulated diseased" panel of standards
Mouse_CCL6 P27784	10.7	9.38	> 97	50	100

Mouse_Lungkine Q9WVL7	16.5	7.07	> 90	10^3^	10^3^

Chicken_Caronte Q9PUK2	56	6.37	> 90	10^4^	10^4^

Mouse_Soggy Q9QZL9	26.8	7.18	> 90	10^5^	3 × 10^5^

Firefly_Luciferase Q27758	120	6.72	10-35	10^6^	6.67 × 10^5^

Chicken-Egg-White_Lysozyme P00698	14.7	9.36	≥ 90	10^7^	10^7^

The "normal" panel of generic standards was used for all work, whereas the "simulated diseased" panel of protein standards was incorporated only in the study to evaluate the ability to identify known changes in composition of the generic standards

Biological test samples were spiked with the QC material, extending over a broad dynamic range (50 - 10^7 ^pg/mL) emulating the natural abundance of proteins in serological samples [11]. If any of these component proteins provide a robust signal independent of biological matrix effects, they may be indicative of a platform being fit-for-purpose, as well as allowing assay performance comparisons between different platforms. Additionally, if a subset of the spike proteins is influenced by matrix effects associated with individual serological samples, this may bring into question the reliability of the detection of the target analytes, which may also be adversely affected by differential matrix effects.

Recombinant (mouse CCL6, mouse lungkine, chicken caronte/Fc chimera, mouse soggy from R&D Systems, Abingdon, UK) and purified proteins (firefly luciferase and chicken egg lysozyme from Sigma, Poole, UK) were reconstituted in PBS prior to gravimetric preparation of the 10× stock QC material (also termed the normal panel of generic standards) and stored at - 80°C. Refer to Additional file 1 for details of characterisation of the homogeneity, Additional file 2 for short-term stability data and Additional file 3 for long-term stability of the QC material.

### MSD assays

Direct MSD^®^-based assays were constructed for each of the six spike proteins and four candidate ovarian cancer biomarkers, either alone or within serological samples. Capture antibodies (rat anti-CCL-6, rat anti-lungkine, rat anti-soggy, rat anti-epidermal growth factor receptor (EGFR), mouse anti-osteopontin, and mouse anti-interleukin-8 (IL-8) monoclonal antibodies (mAbs) and goat anti-caronte polyclonal antibody (pAb) (R&D Systems); anti-luciferase and anti-CA-125 mouse mAbs (AbCam, Cambridge, UK) and mouse anti-lysozyme mAb (Cosmo Bio, Tokyo, Japan) diluted in PBS (at 1-8 μg/mL antibody), were added to wells on either MULTI-ARRAY™ standard bind or high bind MSD^® ^plates (Meso Scale Discovery^®^, Gaithersburg, USA) and incubated overnight at 4°C. The plate was decanted and each well incubated with 150 μL blocking buffer (phosphate-buffered saline (PBS), pH 7.4; 1% (w/v) BSA, 0.05% (w/v) sodium azide) on a shaker for 1 hour at room temperature. After three washes with PBS, 0.05% (w/v) polyoxyethylenesorbitan monolaurate (Tween20), 25 μL of samples and standards (six spike proteins, and human recombinant EFGR/Fc chimera, IL-8, and osteopontin (R&D Systems)) diluted in either blocking buffer, pooled normal human plasma (Firefly Scientific Limited, Manchester, UK), or single donor normal and ovarian cancer diseased plasma (Sera Laboratories International, Haywards Heath, UK) were added to each well and incubated for 1 hour at room temperature on the shaker. After three washes, 25 μL MSD^® ^SULFO-TAG detection antibody (1 or 2 μg/mL; prepared using the manufacturer's protocol) was added to each well and incubated for 1 hour at room temperature on a shaker.

The unreacted SULFO-TAG N-hydroxysuccinimide (NHS)-ester has molecular weight of 1141 Daltons, and after the labelling reaction, the conjugated SULFO-TAG adds 1027 Daltons to the protein. As the SULFO-TAG is a small hydrophilic molecule (approximately 1 kDa), it is not expected to affect the function of large protein conjugation partners such as antibodies, especially as the SULFO-TAG is much smaller than biotin (13 kDa). Biotin NHS-ester is the most commonly used method to label antibodies, which also binds to the antibody via primary amines in the same manner as the SULFO-TAG NHS-ester and is generally not anticipated to interfere with the binding of the antibody to the cognate target antigen.

For labelling, lyophilised antibodies were directly reconstituted in PBS, pH 7.9. For antibodies sourced in solution, buffer exchange was performed with PBS, pH 7.9 using Zeba Desalt Spin columns with a 7000 Dalton molecular weight cut-off threshold (Thermo Scientific). The concentration of the antibody was then determined by the BCA protein assay (Thermo Scientific, Massachusetts, USA) using the microplate procedure. 3 nmol/μL Sulfo-Tag NHS-Ester tag (Meso Scale Discovery) was added to the antibody for the labelling step using a molar challenge ratio of 12:1, and the sample was mixed for 2 hours in the dark at room temperature. The antibodies were then buffer exchanged into PBS, pH 7.4/0.05% sodium azide using Zeba Desalt Spin columns. The concentration of the labelled antibody was then determined by BCA protein assay using the microplate procedure. Absorbance of protein conjugate at 455 nm was measured by NanoDrop (Thermo Scientific) to establish labelling ratio of Sulfo-tag to antibody. The detection antibodies subjected to labelling were goat pAbs: anti-luciferase (Promega, Southampton, UK), anti-caronte, anti-CCL-6, anti-lungkine, anti-soggy, anti-osteopontin, anti-IL-8 and anti-EGFR (R&D Systems). Rabbit detection pAbs were anti-lysozyme (Millipore, Watford, UK), and anti-CA-125 (Bioquote Limited, York, UK). Wells were washed before addition of 150 μL of MSD^® ^Read Buffer T (with surfactant).

A voltage was applied to the carbon electrodes integrated in the plate, initiating a redox reaction involving ruthenium chemistry, resulting in the emission of light detected by the cooled charge coupled device (CCD) camera. The raw data output was analysed by MSD^® ^Discovery Workbench 3.0 software.

### Evaluating the detection of each analyte within the protein mixture

Each of the spike mixtures with five proteins was prepared in pooled normal human plasma, maintaining the designated concentrations for each analyte as outlined in Table 1 for the full complement of proteins. The six combinations of five spike proteins were assayed alongside the full panel of six spike proteins in pooled plasma and the diluent (pooled normal human plasma) as the negative control for each of the six uniplexed assays. Three separate experiments were performed to evaluate the scope of detecting each analyte amidst the complete protein mixture. Triplicate determinants were incorporated within each experiment, and the data for each separate experiment was shown.

### Evaluating the ability to discriminate between two panels with variable abundance in spike proteins within pooled normal human plasma

Two distinct panels of the six protein spikes were prepared, with the composition outlined in Table 1. One panel was termed as the "normal" panel of generic standards. A second panel comprising of 1.5-3 fold changes in the composition of three component proteins (CCL6, soggy and luciferase) with all concentrations remaining within the linear working range of the assay was designated as the "simulated diseased" panel of proteins.

Uniplexed assays for each of the six proteins were performed with both panels of protein standards, incorporating triplicate determinants for all three separate experiments. The reliability of detecting known fold changes in concentrations of selected spike proteins diluted in pooled normal human plasma as the biological matrix, was evaluated by comparing the fold changes between the two protein panels. 7-point internal calibration curves were incorporated for the assay of each analyte.

### Ovarian cancer model spike-in study

Single donor plasma samples (six normal and six ovarian cancer diseased) were supplemented with the QC material to incorporate a 1× working concentration of the six spike proteins and assayed for the six spike proteins and four putative ovarian cancer biomarkers, CA-125, EGFR, IL-8 and osteopontin. Experiments were performed with internal calibration curves (except for CA-125, as the recombinant protein could not be sourced) with triplicate determinants, in a randomised plate format for three separate experiments.

### Data analysis

The MSD Discovery Workbench analysis software version 3.0 was applied to process the data. A 4-parameter logistic model was used for curve-fitting. PCA was performed using SIMCA-P (Umetrics). Linear mixed-effects models were fitted by residual (or restricted) maximum likelihood using the program "R".

## Results

### Evaluating the scope to detect each component protein within the mixture

**Figure 1:**
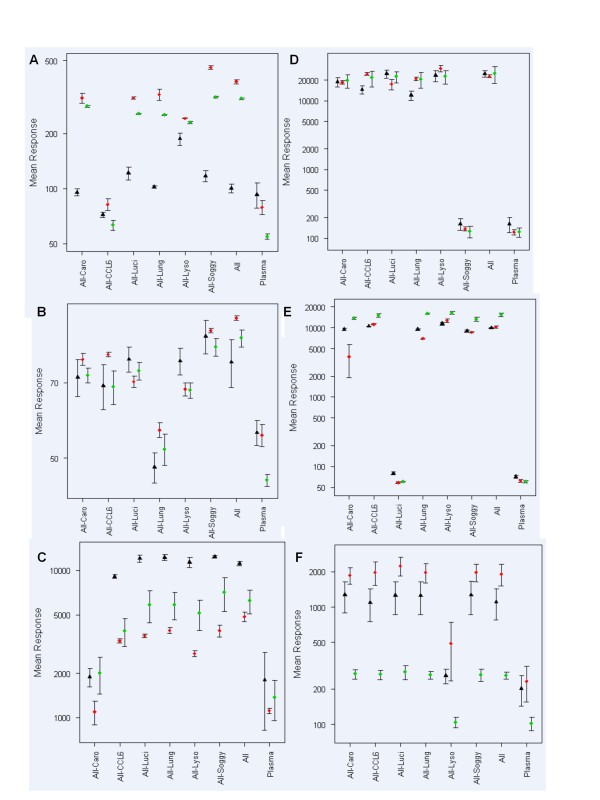
**Evaluation of cross-reactivity associated with the panel of protein standards**. Uniplexed assays for each analyte: (A) CCL6, (B) lungkine, (C) caronte, (D) soggy, (E) luciferase and (F) lysozyme were performed, incorporating the omission of a single protein from the complete mixture of six protein QC material, to evaluate the contribution of each analyte to the overall mean signal output. The data points denote the mean values for each experiment, for day 1 (blue rhombuses), day 2 (pink squares) and day 3 (green triangles), and the error bars represent the SD from triplicate determinants.

Each assay was initially constructed using a background of buffer (blocking buffer) and then pooled normal human plasma as the sample diluent for each analyte, with the criteria that each analyte concentration used is within the linear working range of the assays (data not shown). The contribution to the signal output by each cognate analyte amidst the full panel of proteins was evaluated, to show that the presence of the five non-cognate proteins have no adverse effect on each assay. Each protein individually was removed from the mixture to gauge the contribution by the protein to the overall signal output (Figure 1). A reduction in signal output was anticipated and observed for all assays upon omission of the cognate protein.

### Evaluation of the robustness in detecting each component protein within the spike material comprising of six proteins in pooled normal human plasma

Two distinct panels of protein standards were prepared with the same component proteins but differing (known) concentrations for three proteins; CCL6, soggy and luciferase. The ratios of these three analytes between the two protein panels were experimentally derived from three separate experiments by mean signal outputs and interpolated concentrations from internal calibration curves (Table 2) with the associated standard error of the estimate shown for each analyte within each experiment (Additional file 4). Analysis of data in terms of the signal outputs rather than interpolated values from internal calibration curves produced experimental ratios closest to the theoretical ratios for five out of the six assays. The only exception was the CCL6 dataset; the ratio derived from the interpolated CCL6 concentrations within the two panels more closely resembled the theoretical ratio. This anomalous result with the CCL6 assay was due to the high inter-assay variability in excess of the acceptance criteria of less than 30%, albeit the intra-assay variability was less than 11.5% across all three experiments.

**Table 2:** Detection of compositional fold-changes in selected analytes between two panels of spike proteins

Analyte	Ratio of signal outputs from the "normal" to "simulated diseased" panels of protein standards (n = 3)		Ratio of interpolated concentrations from the "normal" to "simulated diseased" panels of protein standards (n = 3)	
	
	Expected	Actual	Expected	Actual
CCL6	1:2	1: 1.26 ± 0.06	1:2	1: 1.72 ± 0.28

Lungkine	1:1	1: 0.94 ± 0.05	1:1	1: 1.71 ± 1.21

Caronte	1:1	1: 1.11 ± 0.21	1:1	1: 1.20 ± 0.29

Soggy	3:1	3: 1.02 ± 0.11	3:1	3: 0.96 ± 0.11

Luciferase	3:2	3: 1.80 ± 0.19	3:2	3: 1.86 ± 0.43

Lysozyme	1:1	1: 0.96 ± 0.06	1:1	1: 0.88 ± 0.32

The ratios of each analyte between the "simulated normal and diseased" panels of proteins are tabulated below, in terms of signal outputs and interpolated concentrations. Theoretical ratios were calculated based on all analyte concentrations correlating with the linear working range of the assay. Theoretically within the "simulated diseased" panel, the concentration of CCL6 was 2-fold higher, whereas the concentrations of soggy and luciferase were 3-fold and 1.5-fold lower, respectively, compared with the same analytes within the "normal" generic panel of protein standards

Generally, interpolation increased the variability in the ratios between the "normal" and "simulated diseased" panels, with CVs of 5.91 - 70.76%, compared with the CV range of 0.19 - 10.78% associated with the ratios between the two panels derived from the mean signal output data. Hence the material may be used to evaluate the robustness the platform by evaluating the performance of these assays in terms of the signal output, rather than by interpolation from the internal standard curves.

### Implementation of the QC spike protein in a model system

The QC material was spiked into six single donor ovarian cancer plasma samples (termed as OC samples) and six single donor normal plasma samples. Three separate experiments were performed to evaluate the reproducibility of detecting the six spiked analytes, in addition to three putative ovarian cancer biomarkers, IL-8, EGFR, and osteopontin, alongside the FDA-approved marker CA-125. If all four candidate biomarkers are appropriate markers for ovarian cancer, differential expression of these cancer markers is anticipated between the normal and OC samples. It is anticipated that the spiked proteins are detectable at the same abundance if all samples are supplemented with the same quantity of QC material.

**Figure 2:**
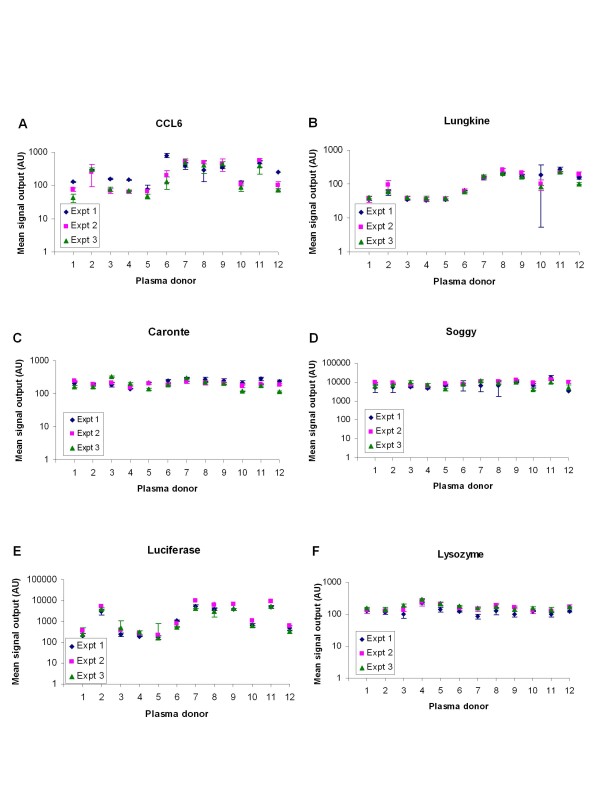
**Evaluation of the utility of each spike protein in single donor plasma samples**. The mean signal output of (A) CCL6, (B) lungkine, (C) caronte, (D) soggy, (E) luciferase and (F) lysozyme from twelve single donor plasma samples supplemented to comprise the 1× stock of the QC material were determined from three separate experiments with n = 3. The data points denote the mean values for experiment 1 (blue rhombuses), experiment 2 (pink squares) and experiment 3 (green triangles), and the error bars represent the SD.

The QC material when spiked into the plasma samples could serve as a useful indicator to verify the robustness of the platform if the signal outputs for its component proteins are reproducible between experimental runs and independent of matrix effects. From the plots of the signal outputs, it was apparent that three proteins, caronte, soggy and lysozyme, fulfilled this criteria (Figure 2). A linear mixed effects model fit by residual (or restricted) maximum likelihood was performed using all data from three separate experiments and the fixed effects are a measure of the statistical difference between the control and diseased plasma samples, based on the signal output observed. The p-values for lysozyme and soggy were 0.175 and 0.189 respectively, which suggests there is no significant difference between the control and cancer plasma samples, across three separate experiments. The p-value for the caronte data was 0.056, which may be indicative of borderline statistical difference between the two sample groups. Sample matrix effects were more apparent for the detection of CCL6, lungkine and luciferase (Figure 2). This led to variable signal outputs across the twelve single donor samples for CCL6, lungkine and luciferase (Figures 2A, B and 2E). With these three spike proteins, there were generally lower signal outputs among the normal samples (donors 1-6) compared with the OC samples (donors 7-12), with the following exceptions: elevated signals for donor 2 and lower signal outputs for donors 10 and 12. However, only the intra-assay and inter-assay CVs of the caronte, soggy, lysozyme, and lungkine assays were typically less than the QC acceptance criteria of 30% variability (Additional file 5). Although the intra-assay variability for luciferase was typically less than 30%, the inter-assay variability was marginally higher than the acceptance criteria. However higher inter-assay variability has been documented for other commercial MSD assays in the context of cytokine assessments (e.g. Chowdhury *et al*., 2009 [12]), in spite of low intra-assay variability. Both the intra- and inter-assay variability of the CCL6 assay were greater than 30%, which suggests that the assay was not sufficiently robust when using the individual plasma donor samples.

**Figure 3:**
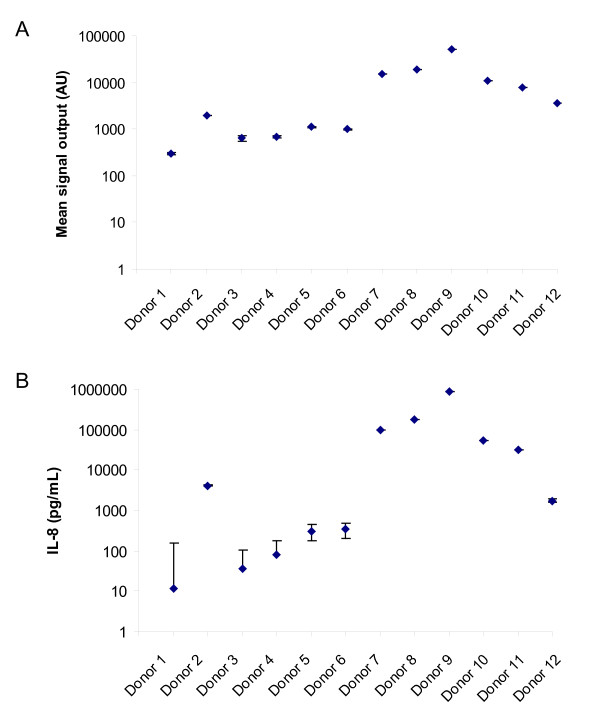
**Evaluation of the reproducibility of IL-8 detection**. IL-8 detection of the twelve single donor plasma samples, are displayed as (A) the mean signal output and as (B) interpolated concentrations of IL-8 from the internal standard curves. These data points (blue rhombuses) are the mean values of triplicate experiments, incorporating triplicate replicate determinants. The error bars denote the SD of the three separate experiments.

Of the four candidate biomarkers, the major contributor to the separation of the data for the two patient populations was IL-8 (data not shown). In addition to evaluating the IL-8 data in terms of the mean signal outputs (Figure 3A) for each experiment, interpolated concentrations of IL-8 detected within the twelve single donor plasma samples were also shown (Figure 3B). Interpolation of data from internal standard curves is a commonly adopted normalisation approach for immunoassays to assign relative concentrations of the analytes of interest within the test samples. Although both methods of analysis indicated some separation between the normal and diseased datasets, it was clear that interpolated IL-8 concentration increased variability observed between experiments with the normal plasma samples. The intra-assay and inter-assay variability for CA-125 and EGFR were generally less than the acceptance criteria of 30% variability (Additional file 5). Anomalous results with the third IL-8 dataset may have contributed to the inter-assay CV of greater than 30%. The osteopontin assays of the individual donor plasma samples were not sufficiently robust as evidenced by the high intra- and inter-assay CVs of more than 30% variability (Additional file 5).

## Discussion

To demonstrate platform robustness, the signal output of the component proteins should not exhibit significant statistical difference between the individual donor plasma samples, or between technical replicates and separate experimental runs. The component proteins exhibiting differential signals between distinct samples would be indicative of the existence of matrix effects that may also influence credibility in the data derived for the detection of the target analytes. In this pilot study, the purpose of the generic QC material was illustrated by immunoassay using a single set of antibodies. These data form a preliminary finding, and all aspects of variability inducing parameters should be subjected to a full validation. A larger scale study with a greater number of individual donor plasma samples, platforms and antibodies may be required to determine the true criteria for acceptable variability in the signal outputs for the component proteins. Generic protein standards have not previously been applied to immunodetection methods, however, it is anticipated that this QC material may be applied to all protein-based detection methods, including mass spectrometry and other immunoassays.

One limitation with immunoassays is the potential for cross-reactivity between proteins and non-cognate antibodies that may impair the detection of the target analyte. We have shown that each protein was detectable amidst the presence of the five non-cognate target proteins. However, with the ovarian cancer pilot study involving single donor plasma samples, the CCL6, lungkine and luciferase assays exhibited differential degrees of cross reactivity, giving rise to the variable signal outputs observed when the QC material was spiked in different patient samples. Interestingly, there was concordance among these three assays in terms of their signal profiles across the twelve donor samples, in spite of the concern regarding the robustness of the CCL6 assay. As this cross-reactivity was not due to the presence of the five other proteins within the spike panel *per se*, it was most probable that the variable protein composition of the biological matrix bound non-specifically to some of the antibodies within these assays.

Lungkine as well as CCL6 and luciferase, at their current designated spike concentrations could not be used as the QC material of platform reliability for immunoassays with single donor plasma samples, with the current set of antibody pairs. However, this does not exclude their use as spike materials using distinct antibody pairs or for assays without antibodies (e.g. mass spectrometry), or as a spike material for other biological samples. These alternative uses will require further investigation on a case-specific basis. Nonetheless, with the existing assay conditions for these three component proteins, their signal outputs are valuable indicators of matrix effects associated with individual samples. Identifying matrix effects is of importance as this phenomenon may also adversely affect the accuracy of quantifying the presence of the target analyte.

Hence, this subset of proteins may collectively highlight the need for caution when evaluating the level of robustness for the test analyte data. Conversely, soggy, lysozyme and possibly caronte do not exhibit sensitivity to sample matrix effects. Hence these latter three proteins may serve as indicators of the robustness of the platform.

Another limitation of many antibody-based assays is the narrow linear range of the working assays, especially when calibrants are spiked into a biological matrix (e.g. plasma) rather than a buffer. Assays may not always exhibit a sufficiently broad linear range encompassing the full concentration coverage for physiological status, leading to interpolation of some data from a non-linear portion of the fitted curve. Albeit curve-fitting within an experiment may be robust, calibration curves may be susceptible to changes in the trendline thus increasing variability between distinct experiments. This subsequently reduces the capacity for robust inter-experiment comparison. High variability with the interpolated analyte concentrations via internal calibration curves from different experimental runs is observed when evaluating the the normal and "simulated disease" protein panels, as well as for IL-8 detection.

In this instance, IL-8 concentration in the OC samples coincided with the linear portion of the IL-8 standard curve, whereas the concentrations of IL-8 associated with normal plasma fell outside this robust range. This brings into question the need for a standard curve, given that detection above an assigned cut-off threshold (e.g. the mean of the normal IL-8 plasma concentration ± 3 SD) may suffice for the diagnosis of disease. The incorporation of internal standard curves also utilises numerous additional wells to ensure there are sufficient datapoints for robust curve fitting, with adequate technical replication. However, using the QC material *in lieu *of internal calibrants for each analyte may demonstrate the platform is fit-for-purpose and improve on assay throughput by reducing the number of wells consumed by calibrants.

## Conclusions

We have shown the utility of the generic protein QC material as an indicator of platform performance and matrix effects, using immunoassays on the MSD Sector 6000 Imager platform. The QC material may also be used alone to evaluate bias introduced by variable instruments, operators or reagents. This panel of proteins has also exhibited suitable homogeneity and stability for this application. The material can be incorporated into a "quality metrics" toolkit for assessing protein biomarker platform performance, addressing issues associated with insufficient assay robustness delineated in the FDA's Critical Path Initiative in 2004.

## Abbreviations

(BSA): Bovine serum albumin; (EGFR): epidermal growth factor receptor; (IL-8): interleukin-8; (mAb): monoclonal antibody; (MSD^®^): Meso Scale Discovery^®^; (MFI): median fluorescent intensity; (OC): ovarian cancer; (pAb): polyclonal antibody; (PBS): phosphate buffered saline; (Tween20): polyoxyethylenesorbitan monolaurate; (QC): quality control.

## Competing interests

The authors declare that they have no competing interests.

## Authors' contributions

SP participated in the experimental design, data interpretation and assisted with the preparation of the gravimetrically prepared protein samples and assays for the ovarian cancer study, and drafted the manuscript. ESA and HJV assisted with the preparation of the gravimetrically prepared protein samples and performed the MSD assays and processed the data using spreadsheets. JM participated in the experimental design and statistical analyses. CAF conceived the study, participated in the interpretation of the data and revision of the manuscript. All authors approved the manuscript.

## Supplementary Material

Additional file 1 **Homogeneity of the QC material**. Ten tubes of the 10× stock QC material were selected randomly from the stock of material stored at -20°C, and three separate dilution steps were performed per tube to evaluate the reproducibility of separate dilution steps. Nested ANOVA was performed to evaluate the variability between distinct tubes and the dilution steps (combined with the variability of the different tubes), as well as the overall variability of the platform. Click here for file

Additional file 2 **Evaluation of the short term stability of the 10× stock QC material**. The isochronous assay for each of the six protein components of the QC material was performed, following exposure of the 10× stock to room temperature, for 1 h, 1 day, 3 days and 7 days prior to the assays. Click here for file

Additional file 3 **Evaluation of the long term stability of the 10× stock QC material**. Uniplexed assays were performed for each the six protein components of the diluted 1× QC material at monthly intervals, following storage of the 10× stock QC material in the -20°C freezer, -80°C freezer, or as a lyophilised powder stored at -20°C. Click here for file

Additional file 4 **Standard error estimated associated to each ratio of normal and simulated diseased signal output for each analyte**. The formula used to calculate the uncertainty associated with the derivation of the ratios between the normal and simulated diseased panels for each analyte is shown. Click here for file

Additional file 5 **Tabulated variability of the assays**. Intra- and inter-assay variability for the 6 spike proteins and 4 candidate ovarian cancer biomarkers derived from three separate experiments conducted to evaluate the implementation of the QC spike protein in a model system. Click here for file
