# Supplementary material for: A pilot study to evaluate the application of a generic protein standard panel for quality control of biomarker detection technologies
Source: BMC Res Notes. 2011 Aug 11;4:281. doi: 10.1186/1756-0500-4-281 (PMC3162916; doi:10.1186/1756-0500-4-281)
Supplement: Additional file 1 — Homogeneity of the QC material. Ten tubes of the 10× stock QC material were selected randomly from the stock of material stored at -20°C, and three separate dilution steps were performed per tube to evaluate the reproducibility of separate dilution steps. Nested ANOVA was performed to evaluate the variability between distinct tubes and the dilution steps (combined with the variability of the different tubes), as well as the overall variability of the platform. [file 1756-0500-4-281-S1.PDF]

Additional file 1

File format: PDF

Homogeneity of the QC material

Ten tubes of the 10x stock QC material were selected randomly from the stock of material stored at -20 ^{o}C, and three separate dilution steps were performed per tube to evaluate the reproducibility of separate dilution steps. Duplicate loadings from each of the subsequent thirty tubes were incorporated for each of the six MSD® assays to gauge the technical reproducibility of the assay in a randomised plate format. The inter-tube variability for all analytes was less than 5.5 %, with the exception of luciferase. Nested ANOVA was performed to evaluate the variability between distinct tubes and the dilution steps (combined with the variability of the different tubes), as well as the overall variability of the platform. These data analyses indicated that the variability between the distinct tubes for each assay was approximately a third of the inherent variability of the platform (i.e. the technical variability), with the exception of the assay for luciferase. With this particular analyte the inter-tube variability was just within the limits of the technical variability of the luciferase assay on this platform. The higher inter-tube variability observed associated with luciferase was perhaps due to the light-sensitivity of this analyte. The contributions by the inter-tube, inter-dilution and technical reproducibility to the total variability exhibited with the detection of each spike protein component of the QC material is shown below.

Spike protein	Uncertainty		
	Tube	Dilution	Replication
CCL6	15 %	34 %	51 %
Lungkine	36 %	15 %	49 %
Caronte	27 %	0 %	73 %
Soggy	33 %	19 %	48 %
Luciferase	47 %	6 %	47 %
Lysozyme	0 %	33 %	67 %
Average	26 %	18 %	56 %

The inter-tube variability for all analytes was less than 5.5 %, with the exception of luciferase which was 9.0 %. The higher inter-tube variability observed associated with luciferase was perhaps due to the light-sensitivity of this analyte. The nested ANOVA shows the contribution to the total uncertainty due to intra-tube variability, the dilution of the 10x stock QC material, and replicate loadings.
