# Supplementary material for: A pilot study to evaluate the application of a generic protein standard panel for quality control of biomarker detection technologies
Source: BMC Res Notes. 2011 Aug 11;4:281. doi: 10.1186/1756-0500-4-281 (PMC3162916; doi:10.1186/1756-0500-4-281)
Supplement: Additional file 2 — Evaluation of the short term stability of the 10× stock QC material. The isochronous assay for each of the six protein components of the QC material was performed, following exposure of the 10× stock to room temperature, for 1 h, 1 day, 3 days and 7 days prior to the assays. [file 1756-0500-4-281-S2.PDF]

Additional file 2

File format: PDF

Evaluation of the short term stability of the 10x stock QC material

The isochronous assay for each of the six protein components of the QC material was performed, following exposure of the 10x stock to room temperature, for 1h, 1 day, 3 days and 7 days prior to the assays. Each datum point represents the mean value of the signal output from three separate dilutions from each stock tube of QC material, and the data for all three tubes per time point is displayed.

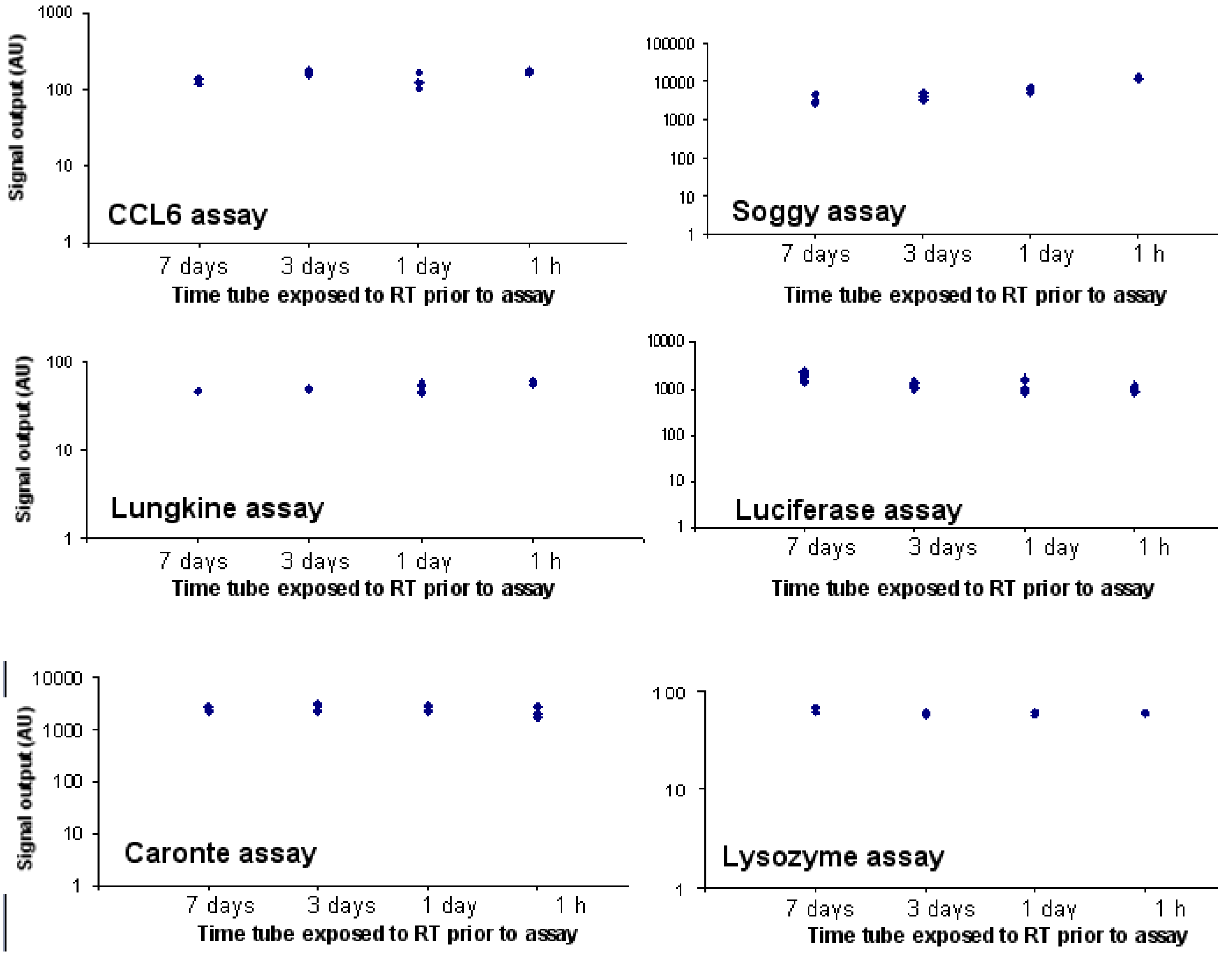

The purpose of the evaluation of the short term stability was to emulate the effects of transit of the QC material; there was no adverse effect on the stability of the protein mixture when exposed for up to 7 days at room temperature.
