# Supplementary material for: A pilot study to evaluate the application of a generic protein standard panel for quality control of biomarker detection technologies
Source: BMC Res Notes. 2011 Aug 11;4:281. doi: 10.1186/1756-0500-4-281 (PMC3162916; doi:10.1186/1756-0500-4-281)
Supplement: Additional file 3 — Evaluation of the long term stability of the 10× stock QC material. Uniplexed assays were performed for each the six protein components of the diluted 1× QC material at monthly intervals, following storage of the 10× stock QC material in the -20°C freezer, -80°C freezer, or as a lyophilised powder stored at -20°C. [file 1756-0500-4-281-S3.PDF]

Additional file 3

File format: PDF

Evaluation of the long term stability of the 10x QC material

Uniplexed assays were performed for each the six protein components of the diluted 1x QC material at monthly intervals, following storage of the 10x stock QC material in the -20 ^{o}C freezer (blue diamond), -80 ^{o}C freezer (red triangle), or as a lyophilised powder stored at -20 ^{o}C (pink triangle). Each datum point represents the mean value of the signal output for individual dilutions from stock tubes of QC material from each storage condition evaluated.

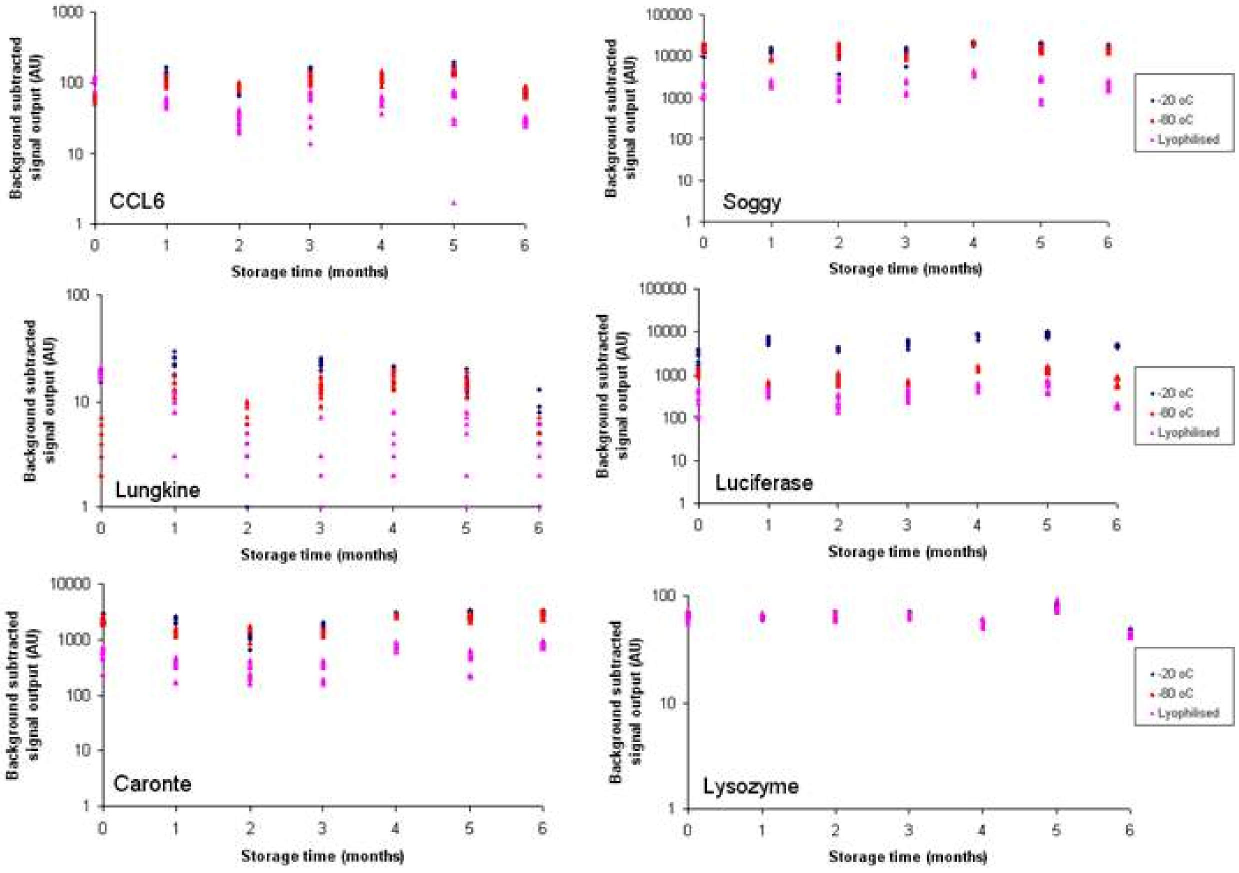

It was apparent that a stable signal output could be observed for five of the six analytes, with the only exception being lungkine which did not produce sufficient signal output. Overall, the lyophilised protein gave rise to a broader spread in the signal output for most assays, when comparing with the storage of frozen protein solution at -20 ^{o}C and - 80 ^{o}C. Interestingly, the results from the analysis of protein solution stored at -20 ^{o}C at -80 ^{o}C are comparable, and that all proteins except lungkine were stable throughout the six months of storage.
