# Supplementary material for: A pilot study to evaluate the application of a generic protein standard panel for quality control of biomarker detection technologies
Source: BMC Res Notes. 2011 Aug 11;4:281. doi: 10.1186/1756-0500-4-281 (PMC3162916; doi:10.1186/1756-0500-4-281)
Supplement: Additional file 4 — Standard error estimated associated to each ratio of normal and simulated diseased signal output for each analyte. The formula used to calculate the uncertainty associated with the derivation of the ratios between the normal and simulated diseased panels for each analyte is shown. [file 1756-0500-4-281-S4.PDF]

Additional file 4

File format: PDF

Standard error estimated associated to each ratio of normal and simulated diseased signal output for each analyte

The uncertainty associated with the derivation of the ratios between the normal and simulated diseased panels for each analyte has been represented as the standard error estimated for each experiment. The standard error of the estimate (SEE) of the ratio is calculated as per formula stated below:

$$SEE(ratio) = \sqrt{\left(\frac{u(normal)}{\overline{N}}\right)^2 + \left(\frac{u(diseased)}{\overline{D}}\right)^2}$$

Where $\overline{N}\, and\, \overline{D}$ are the means of the three replicates performed in each experiment.

The standard uncertainties *u(normal) and u(diseased)* are calculated as follows:

$$u(normal) = \frac{SD(normal)}{\sqrt{n}}$$

Where n=3 (number of replicates)

The SEE is the sum of variance components.

Analyte	Expt	Normal	Diseased	SD_{normal}	$SD_{diseased}$	Ratio	SEE
Caronte	1	135.667	129.667	6.658	7.767	1.046	0.045
Caronte	2	261.667	263.667	7.767	11.372	0.992	0.030
Caronte	3	125.667	170.000	20.648	1.000	0.739	0.095
CCL6	1	116.333	138.667	5.508	4.619	0.839	0.033
CCL6	2	64.333	83.667	0.577	5.132	0.769	0.036
CCL6	3	64.333	83.000	1.155	2.000	0.775	0.017
Luciferase	1	863.000	476.667	51.420	30.925	1.810	0.051
Luciferase	2	859.000	568.000	82.940	105.702	1.512	0.121
Luciferase	3	1207.333	698.667	32.021	4.163	1.728	0.016
Lungkine	1	44.333	39.333	5.686	2.309	1.127	0.081
Lungkine	2	46.667	46.333	2.082	5.686	1.007	0.075
Lungkine	3	49.000	46.333	2.646	1.528	1.058	0.037
Lysozyme	1	215.000	196.000	5.292	9.165	1.097	0.031
Lysozyme	2	239.333	244.333	10.693	17.039	0.980	0.048
Lysozyme	3	203.000	193.333	2.000	14.572	1.050	0.044
Soggy	1	17043.667	5542.000	713.318	1102.907	3.075	0.117
Soggy	2	9330.667	2858.667	935.116	207.760	3.264	0.071
Soggy	3	11305.000	4253.000	121.618	201.099	2.658	0.028
