# Supplementary material for: A pilot study to evaluate the application of a generic protein standard panel for quality control of biomarker detection technologies
Source: BMC Res Notes. 2011 Aug 11;4:281. doi: 10.1186/1756-0500-4-281 (PMC3162916; doi:10.1186/1756-0500-4-281)
Supplement: Additional file 5 — Tabulated variability of the assays. Intra- and inter-assay variability for the 6 spike proteins and 4 candidate ovarian cancer biomarkers derived from three separate experiments conducted to evaluate the implementation of the QC spike protein in a model system. [file 1756-0500-4-281-S5.PDF]

Additional file 5

File format: PDF

Tabulated variability of the assays

Intra- and inter-assay variability for the 6 spike proteins and 4 candidate ovarian cancer biomarkers derived from three separate experiments conducted to evaluate the implementation of the QC spike protein in a model system. CVs above the QC acceptance criteria of 30% are highlighted in red.

Assay	Plasma	Intra-assay CV			Inter-assay CV
		Expt 1	Expt 2	Expt 3	
Caronte	Donor 1	12.50	3.04	5.75	19.94
Caronte	Donor 2	4.70	7.92	5.18	11.98
Caronte	Donor 3	15.22	7.97	4.28	30.66
Caronte	Donor 4	1.62	8.79	6.39	19.63
Caronte	Donor 5	6.00	5.28	5.84	22.83
Caronte	Donor 6	14.00	9.13	6.60	15.83
Caronte	Donor 7	14.59	12.79	2.93	11.79
Caronte	Donor 8	13.65	14.56	4.18	13.54
Caronte	Donor 9	12.44	9.67	4.41	13.23
Caronte	Donor 10	14.85	13.06	2.91	29.23
Caronte	Donor 11	10.31	2.80	6.81	25.93
Caronte	Donor 12	10.09	8.45	4.40	33.65
Soggy	Donor 1	47.26	4.34	14.05	34.02
Soggy	Donor 2	50.26	10.73	6.11	23.99
Soggy	Donor 3	7.72	9.96	15.48	32.03
Soggy	Donor 4	11.35	2.37	18.87	18.70
Soggy	Donor 5	8.60	8.80	16.89	34.20
Soggy	Donor 6	55.45	6.75	14.86	0.77
Soggy	Donor 7	51.58	7.59	16.44	29.82
Soggy	Donor 8	73.89	15.39	19.15	24.16
Soggy	Donor 9	8.45	4.03	9.51	13.20
Soggy	Donor 10	21.05	25.44	12.87	36.26
Soggy	Donor 11	28.35	5.25	16.81	27.97
Soggy	Donor 12	129.99	18.57	30.07	55.00

Lysozyme	Donor 1	3.15	20.25	4.58	9.75
Lysozyme	Donor 2	12.59	11.39	13.95	9.29
Lysozyme	Donor 3	23.64	15.50	9.37	30.83
Lysozyme	Donor 4	8.00	22.18	4.44	14.73
Lysozyme	Donor 5	17.78	10.17	14.56	22.45
Lysozyme	Donor 6	4.61	3.06	5.44	19.01
Lysozyme	Donor 7	14.84	15.83	4.52	29.50
Lysozyme	Donor 8	25.61	18.47	14.27	17.21
Lysozyme	Donor 9	19.09	9.64	14.01	23.10
Lysozyme	Donor 10	3.65	15.14	12.25	11.66
Lysozyme	Donor 11	17.18	4.97	20.24	15.18
Lysozyme	Donor 12	5.71	13.88	2.56	17.98
CCL6	Donor 1	2.77	11.62	26.96	51.91
CCL6	Donor 2	9.42	63.23	10.94	8.40
CCL6	Donor 3	6.95	16.96	13.50	47.70
CCL6	Donor 4	3.20	8.84	3.03	49.87
CCL6	Donor 5	26.53	17.21	15.28	24.56
CCL6	Donor 6	109.48	37.20	40.31	97.06
CCL6	Donor 7	90.43	8.92	26.95	13.23
CCL6	Donor 8	156.39	7.75	44.07	26.33
CCL6	Donor 9	35.74	40.70	15.43	12.67
CCL6	Donor 10	12.89	15.49	26.87	15.72
CCL6	Donor 11	112.18	18.83	43.63	18.91
CCL6	Donor 12	1.56	27.61	9.50	68.15
Lungkine	Donor 1	5.71	20.84	7.70	7.15
Lungkine	Donor 2	20.61	33.70	10.49	30.26
Lungkine	Donor 3	0.00	13.13	10.02	6.68
Lungkine	Donor 4	5.25	13.09	15.56	7.12
Lungkine	Donor 5	3.33	12.37	6.78	5.41
Lungkine	Donor 6	2.40	17.86	3.94	4.08
Lungkine	Donor 7	12.23	3.54	13.01	4.84
Lungkine	Donor 8	6.11	10.66	6.63	15.68
Lungkine	Donor 9	10.37	9.86	8.96	16.81
Lungkine	Donor 10	97.33	34.62	12.16	45.39
Lungkine	Donor 11	15.59	6.34	3.09	14.06
Lungkine	Donor 12	6.83	12.58	6.49	31.77
Luciferase	Donor 1	27.72277	26.91	5.08	29.26
Luciferase	Donor 2	29.56	1.71	33.10	27.88
Luciferase	Donor 3	21.26	9.71	5.08	34.19
Luciferase	Donor 4	0.78	8.63	3.90	20.34
Luciferase	Donor 5	21.16	36.05	9.96	18.01
Luciferase	Donor 6	4.95	12.53	12.26	34.26
Luciferase	Donor 7	21.13	13.29	6.18	48.90

Luciferase	Donor 8	12.71	30.00	20.54	40.42
Luciferase	Donor 9	13.13	9.21	14.40	35.58
Luciferase	Donor 10	18.86	7.10	8.28	31.68
Luciferase	Donor 11	16.30	2.48	11.49	33.90
Luciferase	Donor 12	26.24	11.38	5.16	29.81
EGFR	Donor 1	0.91	4.86	40.17	16.14
EGFR	Donor 2	8.97	16.21	3.58	25.83
EGFR	Donor 3	7.88	6.24	3.73	34.95
EGFR	Donor 4	6.23	4.51	3.13	22.23
EGFR	Donor 5	15.62	1.32	3.98	27.80
EGFR	Donor 6	26.74	9.17	4.23	28.20
EGFR	Donor 7	16.53	6.40	1.84	31.66
EGFR	Donor 8	4.12	12.61	15.39	23.13
EGFR	Donor 9	30.08	6.96	2.72	27.57
EGFR	Donor 10	12.88	12.21	6.45	34.52
EGFR	Donor 11	12.20	19.17	10.47	31.74
EGFR	Donor 12	24.61	6.03	3.47	25.07
CA125	Donor 1	2.39	8.63	2.32	19.49
CA125	Donor 2	11.90	2.31	13.84	24.06
CA125	Donor 3	13.68	1.02	36.79	14.73
CA125	Donor 4	2.80	13.00	11.15	3.67
CA125	Donor 5	6.97	5.30	34.30	6.33
CA125	Donor 6	2.11	2.44	6.75	26.61
CA125	Donor 7	10.68	14.04	21.14	38.29
CA125	Donor 8	4.21	8.18	4.81	20.79
CA125	Donor 9	10.86	7.30	6.47	17.46
CA125	Donor 10	16.94	6.40	4.79	12.19
CA125	Donor 11	6.78	2.06	14.77	18.96
CA125	Donor 12	12.19	6.57	13.12	0.98
IL8	Donor 1	15.26	18.96	36.19	19.77
IL8	Donor 2	23.54	21.54	38.50	10.78
IL8	Donor 3	3.87	2.93	51.41	81.77
IL8	Donor 4	3.23	7.63	9.67	47.59
IL8	Donor 5	2.45	5.98	21.91	53.51
IL8	Donor 6	19.50	4.19	7.86	25.19
IL8	Donor 7	18.58	12.94	73.67	26.58
IL8	Donor 8	6.18	38.88	62.70	23.29
IL8	Donor 9	26.16	3.43	66.56	29.64
IL8	Donor 10	20.77	9.53	83.54	29.35
IL8	Donor 11	12.28	5.12	40.82	19.13
IL8	Donor 12	9.75	5.10	78.45	61.48
Osteopontin	Donor 1	65.19	5.40	1.37	63.78
Osteopontin	Donor 2	81.16	85.45	84.19	18.05

Osteopontin	Donor 3	15.02	25.29	82.80	17.76
Osteopontin	Donor 4	56.54	6.81	148.81	47.73
Osteopontin	Donor 5	2.82	29.90	164.21	42.96
Osteopontin	Donor 6	83.46	11.41	12.41	62.05
Osteopontin	Donor 7	75.59	5.28	23.86	12.01
Osteopontin	Donor 8	28.52	2.82	9.04	78.31
Osteopontin	Donor 9	7.18	28.58	8.61	14.34
Osteopontin	Donor 10	8.01	34.62	80.69	24.00
Osteopontin	Donor 11	79.19	13.47	84.31	35.78
Osteopontin	Donor 12	78.67	26.60	147.53	54.36
